# Design of a school-based randomized trial to reduce smoking among 13 to 15-year olds, the X:IT study

**DOI:** 10.1186/1471-2458-14-518

**Published:** 2014-05-28

**Authors:** Anette Andersen, Lotus Sofie Bast, Lene Winther Ringgaard, Louise Wohllebe, Poul Dengsøe Jensen, Maria Svendsen, Peter Dalum, Pernille Due

**Affiliations:** 1 National Institute of Public Health, University of Southern Denmark, Øster Farimagsgade 5A, 1353 Copenhagen, Denmark; 2 Department for Prevention and Documentation, The Danish Cancer Society, Strandboulevarden 49, 2100 Copenhagen, Denmark

**Keywords:** Smoking prevention, Adolescence, School-based, Parental involvement, Randomized trial, Design, Intervention

## Abstract

**Background:**

Adolescent smoking is still highly prevalent in Denmark. One in four 13-year olds indicates that they have tried to smoke, and one in four 15-year olds answer that they smoke regularly. Smoking is more prevalent in socioeconomically disadvantaged populations in Denmark as well as in most Western countries. Previous school-based programs to prevent smoking have shown contrasting results internationally. In Denmark, previous programs have shown limited or no effect. This indicates a need for developing a well-designed, comprehensive, and multi-component intervention aimed at Danish schools with careful implementation and thorough evaluation.

This paper describes X:IT, a study including 1) the development of a 3-year school-based multi-component intervention and 2) the randomized trial investigating the effect of the intervention. The study aims at reducing the prevalence of smoking among 13 to 15-year olds by 25%.

**Methods/Design:**

The X:IT study is based on the Theory of Triadic Influences. The theory organizes factors influencing adolescent smoking into three streams: cultural environment, social situation, and personal factors. We added a fourth stream, the community aspects. The X:IT program comprises three main components: 1) smoke-free school premises, 2) parental involvement including smoke-free dialogues and smoke-free contracts between students and parents, and 3) a curricular component. The study encompasses process- and effect-evaluations as well as health economic analyses. Ninety-four schools in 17 municipalities were randomly allocated to the intervention (51 schools) or control (43 schools) group. At baseline in September 2010, 4,468 year 7 students were eligible of which 4,167 answered the baseline questionnaire (response rate = 93.3%).

**Discussion:**

The X:IT study is a large, randomized controlled trial evaluating the effect of an intervention, based on components proven to be efficient in other Nordic settings. The X:IT study directs students, their parents, and smoking prevention policies at the schools. These elements have proven to be effective tools in preventing smoking among adolescents. Program implementation is thoroughly evaluated to be able to add to the current knowledge of the importance of implementation. X:IT creates the basis for thorough effect and process evaluation, focusing on various social groups.

**Trial registration:**

Current Controlled Trials ISRCTN77415416.

## Background

Smoking is still by far the most health-compromising risk behavior. Smoking is, in Denmark, the cause of every fourth death annually and although smokers live shorter lives, they may expect more years with long-standing diseases [1]. The prevalence of adolescent smokers in Denmark has been decreasing over the past two decades. However, since 2006, the trend seems to have leveled out, especially among boys [2,3]. In Denmark, one fourth of the 13-year olds and almost half of the 15-year olds have tried to smoke. Eight percent of the 13-year olds smoke on a regular basis. Twenty-three percent of the 15-year-old boys and 24% of the girls report that they smoke on a daily, weekly, or monthly basis [4]. Smoking is more prevalent among Danish adolescents from lower socioeconomic backgrounds (SEP) [5,6]. This socioeconomic pattern is recognized in most Western countries [7].

Danish legislative initiatives in 2001 and 2004 seem to have lowered the prevalence of young smokers. In 2001, a national law concerning smoke-free environments banned student’s smoking in Danish public schools. This law was revised in 2007 to include the teachers, who were no longer allowed to smoke on school premises unless in special smoking areas with restricted access. In 2004, the first age limit for buying cigarettes was introduced at age 16. The prevalence of regular smoking among girls was almost halved during the years 1998 to 2006; among 13-year olds from 15% to 7%; and among 15-year olds from 38% to 25%. Among 13-year-old boys, the prevalence of regular smoking fluctuated around 11%, but among 15-year olds it decreased from 31% to 27% [8]. From 2006 to 2010, the prevalence of 13- and 15-year-old smokers in Denmark did not change, although the age limit for buying cigarettes was raised to 18 years in 2008 [4]. Thus, to further reduce smoking prevalence among young Danes, it is necessary to supplement current legislative initiatives with other means of intervention.

School-based programs for smoking prevention have been widely used internationally, but evaluations have shown contrasting results [9]. Comprehensive strategies using a number of extensive components are generally more effective than information-based interventions only, which have shown limited or no effect [10-12]. Between 1998 and 2001, Denmark participated in the international intervention study “The European Smoking Prevention Framework Approach” (ESFA). ESFA was a community-based, randomized controlled trial, which targeted four intervention levels, i.e. adolescents in school, school policies, parents, and the community. Overall, ESFA showed a small but significant effect with a 6% lower increase in weekly smokers in the experimental group over a 30 month period. ESFA showed the strongest effect in Portugal and smaller effects in Finland and Spain. Unfortunately, ESFA did not decrease adolescent smoking in Denmark, the Netherlands, or the UK [13]. Two nation-based interventions in Denmark, “Smoke-free classes” and “Tackling” did not show to have an effect either [14]. A well designed comprehensive, multi-component intervention approach, with careful implementation leading to high engagement among participants, and followed by thorough implementation and evaluation, is therefore required in Denmark.

We developed a school-based, multi-component program for the prevention of adolescent smoking, X:IT. The aims of this article are: 1) to describe the development of the X:IT intervention, 2) to examine, to which degree randomization of the trial resulted in comparable groups of intervention and control schools, and 3) to describe the evaluation of the intervention.

## Methods/Design

The overall aim of the X:IT study was to develop, implement, and evaluate an intervention, easily applicable and sustainable in schools and municipalities.

### The theoretical model

The Theory of Triadic Influences (TTI) encompasses the general cultural environment in which adolescents mature, the more immediate social situation in which they find themselves, and intrapersonal differences among adolescents. These three streams of influence work through different mediating variables, e.g. attitudes, normative beliefs, and self-efficacy. They finally affect the outcomes, smoking intentions, and smoking behaviour [15]. TTI was developed as a theoretical framework to capture influences on experimental tobacco and alcohol use. It has found use in previous studies of smoking in adolescence [16-18] and in studies of other health behaviors, e.g. increased fruit and vegetable intake in the “Pro Children project” [19]. As was the case in the “Pro Children project,” we separated the cultural environment stream into two streams of influence: attitudinal influences, which capture the close cultural environment of the adolescents; and environmental influences, which capture the structural aspects that are known to influence adolescent smoking.

**Figure 1:**
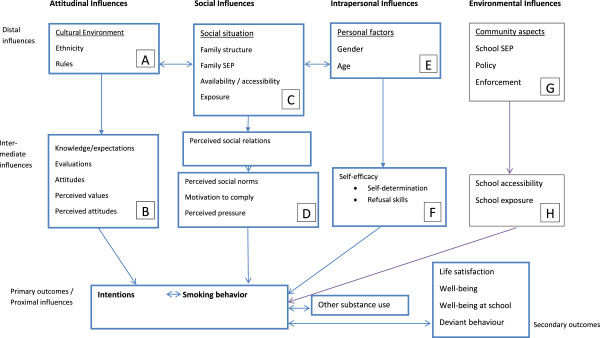
**Theoretical model for the X:IT project based on the Theory of Triadic Influences.** The letters A to H refer to the intermediate factors in the pathway model (Figure 2).

Using TTI and a social ecological approach [20], we developed the conceptual model for the X:IT study (Figure 1).

The Intervention Mapping guidelines were used to inspire the development of the study [21]. Reporting of this study’s findings will comply with the CONSORT statement for cluster randomized controlled trials [22,23].

### Development of the intervention

First, we conducted a thorough literature review on determinants of smoking initiation to uncover determinants at different levels. Second, we conducted a comprehensive search of the literature to review existing interventions shown to be effective. The search led us to focus on the Norwegian program “Fri” [24] and the Swedish program “Tobacco Free Duo” [25]. Since these countries have a culture resembling the Danish their positive experiences might be transferable to a Danish context. Overall, it was decided that X:IT should include three main intervention components: 1) completely smoke-free school premises; 2) parental involvement comprising two dimensions: a) a smoke-free contract between the student and an adult person, preferably a parent, b) smoke-free dialogues; and 3) a smoke-free curriculum based on self-efficacy training and outcome expectancies.

### Intervention components

#### Smoke-free school premises

Danish legislation against smoking on school premises (before August 2012) meant that students and teachers were not allowed to smoke on school premises during school hours. However, schools were allowed to have an indoor smoking area for teachers, provided it was not located nearby students.

The association between teachers smoking during school hours and students smoking has been examined in the literature. A Danish study showed that students exposed to teachers’ smoking on the outdoor school premises were more likely to smoke daily, taking relevant confounders into account [26]. Recent studies from the US showed that higher levels of perceived enforcement of anti-smoking policy at the school level were inversely associated with the prevalence of the past-30-day smoking behaviors among students, independent of individual-level predictors [27,28]. Based on these observations, the X:IT intervention was defined to require smoke-free premises indoors as well as outdoors. This requirement applied to both students and teachers throughout school hours. Schools were encouraged to plan enforcement strategies.

After pilot testing, it became obvious that in order to include a sufficient number of schools, it was necessary to allow some schools to make an exception from the intervention requirement of totally smoke-free school premises. The revised schedule accepted schools with outdoor school premises to be used for teachers smoking. However, teacher smoking during school hours was required to be invisible to students (22 of 43 control schools and 26 of 51 intervention schools had smoking areas for teachers). For students, schools were still required to be completely smoke-free on all school premises during school hours.

#### Parental involvement

Both the Norwegian study ‘FRI’ and the Swedish study ‘Tobacco Free Duo’ have successfully used smoke-free contracts between students and parents. In the Swedish study, this component separately appeared to reduce smoking prevalence by almost 50% [24]. By signing a smoke-free contract, the adolescent promises to stay smoke free for the next year. Signing the contract is a manifestation of an active choice of non-smoking. One of the parents or another adult co-signs the contract. With the contract, the signatory promises to conduct a smoke-free dialogue with the adolescent and to support the adolescent’s choice of staying smoke-free. Having a smoke-free dialogue involves that the parent clearly takes exception to adolescent smoking, and that the parent asks the child about thoughts about and experiences with tobacco. This kind of constructive communication has shown to be effective [29]. The adolescents are motivated to make a personal choice, and engaging the parents signals a clear opposition to adolescent smoking. Smoke-free contracts and smoke-free dialogues are part of the X:IT intervention. Students who remained smoke-free for one year were able to win a prize, sponsored by the municipalities.

#### Smoke-free curriculum

A Cochrane review concluded that there is no strong evidence for the effect of school-based programs that provide information-giving curriculum components only. On the other hand, an information-giving curriculum incorporated in multi-modal programs seem to be successful [9]. Programs based on social influence approaches which included: 1) correcting adolescents’ perceptive overestimation of the smoking prevalence; 2) recognizing high-risk situations, 3) increasing awareness of media, peer, and family influences; 4) teaching and practicing refusal skills; and 5) making public commitments not to smoke, were more effective [9,30]. The teaching program for the X:IT intervention was developed based on the above awareness. The program includes eight lessons a year for three years holding detailed study guidelines for each educational year. The teachers can choose methods of teaching as well as supplementary exercises and materials.

**Figure 2:**
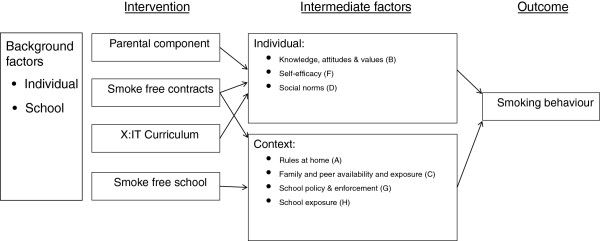
**Model of expected pathways of program effect pathways for the X:IT study**. The letters A to H refer to factors in the influential streams in Figure 1.

The actual educational material, “Gå op I røg” (Up in Smoke), was developed in conjunction with scholars who had educational experience. The material targets students, 13 to 15 years of age. It is designed to be used in diverse subjects such as science, humanities, and social science. Goals in the National Executive Order of Education can be fulfilled by using the material. The material is organized as two books for pupils and two books for teachers.A model of expected pathways for effects of the X:IT program is provided in Figure 2. The figure shows influences of each intervention component and the intermediate individual and contextual factors they are expected to influence on the way to affecting the outcome.

Other materials and tools were developed for 1) municipalities and schools. These materials and tools included background information and scientific documentation, information pamphlets concerning the study and the teaching material, detailed guidelines, and check lists. Furthermore, templates for letters, graphic tools, e.g. logos for mails and letters, newsletters, and a Power Point presentation eligible for introducing the study at parent-meetings at the schools. 2) for parents: smoke-free contracts, pamphlets, and a homepage, http://www.roegfridialog.dk.

All materials and information are available at the X:IT homepage, http://www.xit-web.dk. An overview of all intervention components of the X:IT study is presented in Table 1.

**Table 1:** The intervention components of the X:IT study

***Setting/arena***	***Intervention component***	***Timing***	***Determinant***	***Learning objective***
*Class*	Smoke free curriculum	At least 8 lessons a year in year 7, 8, and 9	Knowledge	• Increase awareness of long- and short-term risks of smoking
				• Reduce ‘majority misunderstanding’
				• Increase awareness of smoking inducing mechanism in the society
			Self-efficacy	• Increase individual ability to resist temptation to smoke
			Perceived norms and attitudes	• Increase identification with non-smokers
				• Contribute to creating smoke free environments
*Home/parents*	Smoke free contract	Start of every school term	Parental attitudes	• Create supportive smoke free environment
				• Signal opposition to adolescent smoking
			Availability	• Reduce availability of cigarettes
			Exposure	• Reduce exposure to smoking
			Perceived norms and attitudes	• Increase identification with non-smokers
				• Contribute to create smoke free environments
	Leaflet for parents	Start of every school term		• Inform about the study, especially the smoke free contract
*School*	Smoke free school	Throughout study	Exposure	• Remove exposure to smoking
			Perceived norms and attitudes	• Increase identification with nonsmokers
				• Contribute to creating smoke free environments
	Parent-teacher meetings	Start of every school term		• Present study information, especially the smoke free contract
*School and municipality*	Kick-off meetings for coordinators at intervention schools and in municipalities	Before start of study period, spring 2010		• Inform about background and methods in the study
	1 day workshops for coordinators at intervention schools and in municipalities	Spring 2011, 2012 and 2013		• Share experiences and receive inspiration to solve problems
				• Get inspired to new teaching methods
				• Secure sustainability of the study
	Newsletters for intervention- and control schools, and municipalities	3-4 each year throughout study		• Inform about study
				• Secure sustainability of the study
	Study reports for each school and municipality	Spring 2011, 2012 and 2013		• Inform about prevalences from the study
				• Secure sustainability of the study
*Municipality*	Sponsoring smoke free student prize	Spring 2011, 2012 and 2013		• Smoke free competition

### Pilot testing

In the autumn of 2008, staff in charge of health promotion at the 98 municipalities in Denmark received an invitation to participate in a pre-conference about the X:IT study. The aim of this workshop was to inform municipalities about the study and to receive feed-back on an early draft of the design of the intervention. One hundred and twelve individuals from 47 municipalities participated. Discussions at the conference led to several changes in the program. One of the early main components -local non-smoking clubs known from the Swedish program “SMART” – was considered ethically inappropriate in a Danish setting and was left out of the intervention.

The intervention was pilot tested in two municipalities and ten schools in one of the municipalities. Qualitative data were collected from project coordinators in the municipalities, from headteachers, teachers, pupils and parents. Results from the pilot tests showed an overall satisfaction with the program. As described earlier, the pilot test demonstrated a need to relax the very strict nonsmoking school policies that were part of the initial program. In the pilot test, the educational material was described to be of high quality, easy to use, and inviting. As schools are generally pressed for time, the material was designed to be part of the mandatory curriculum, and the pilot test emphasized this to be an advantage.

Study coordinators at the municipalities and headteachers at the schools emphasized that economic resources and precise indication of expected time expenditure were important for their acceptance of the study. Also, they found it essential to have centralized management of the study at the municipal level and to have local coordinators at schools and at municipalities. Coordinators, teachers, and parents emphasized that detailed guidelines for all tasks would be useful. Furthermore, they stressed the need of reminders about the study during the school year. This was included in the final program as newsletters.

Lottery prizes in relation to the contracts were appreciated as important motivational factors for the students. In the final program, the prizes were prioritized accordingly.

In the spring 2010, baseline questionnaires for students were pilot tested in five year 7 classes at two socioeconomically diverse schools in the municipality of Aalborg. The students answered the questionnaires and focus group interviews were subsequently conducted with some of the pupils. Only minor revisions were necessary after testing among students.

A newsletter was provided twice a year for municipalities, intervention and control schools taking part in the study. Updates on materials and guidelines are sent out via emails and are available on the X:IT webpage.

### Evaluation

#### Design

The X:IT study will be evaluated by means of effect, process, and health economic evaluations. We used a cluster randomized controlled design for the effect evaluation of the intervention.

#### Setting

Denmark has 98 municipalities with an average 50,000 to 60,000 inhabitants. According to Eurostat, Denmark is one of the least urbanized countries in Europe with only 22% of the population living in urban regions compared to 41% on average in all European countries (European Commission http://epp.eurostat.ec.europa.eu/cache/ITY_PUBLIC/1-30032012-BP/EN/1-30032012-BP-EN.PDF). There would be around one school pr. 5,000 inhabitants in a municipality. The Danish public school consists of year 0 (preschool class) and year 1-9. All 10 years are mandatory. The Danish children start school the year they turn 6. Children who start together in the same class at year 0 will belong to the same class/group of children through all ten years of schooling. There is a limit of 28 children per class. Schools with year 7-9 students have 2-4 parallel tracks. There is no grouping by ability in the Danish schools i.e. that all children have joint lecturing. 85% of all Danish children attend the public school and the schools are area-based, which means that they have a wide socioeconomic composition.

#### Population

**Figure 3:**
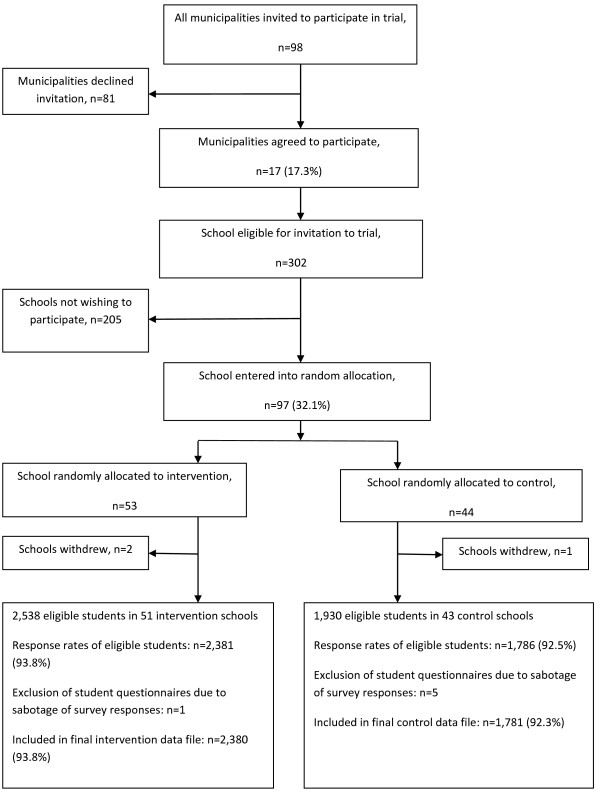
Flow diagram of recruitment, randomization, and participation of municipalities, schools and students at baseline in the X:IT study.

Seventeen municipalities of the 98 invited agreed to participate in the study. All selected a municipal coordinator of the study. The municipalities in the X:IT study varies between 35,000 and 295,000 inhabitants, and included five of the ten largest municipalities in Denmark. They are spread all over Denmark.According to the study plan, municipal coordinators were to address and recruit schools from their municipality. However, due to a challenging recruitment process, research staff had to assist the coordinators. Within the 17 municipalities, 97 of 302 eligible schools agreed to participate in the study and all of these selected a school coordinator for the study (Figure 3).

#### Randomization

Randomization of schools was stratified within the 17 municipalities. Schools recruited were randomized to either the control or intervention group by drawing lots. Fifty-three schools were randomized to intervention schools and 44 to control schools. Three schools withdrew after randomization, leaving 51 interventions schools and 43 control schools in the final study (Figure 3).

#### Implementation

Implementation was launched by one-day kick-off workshops for school head teachers, school coordinators, and municipal coordinators in the spring of 2010. Staff from the Danish Cancer Society, who developed the intervention and the teaching material, led the meetings. The kick-off workshop included a thorough presentation of the intervention and the study, its aim and background, an introduction to the teaching material, and an overview of the evaluation of the study. During the workshop, participants were supposed to make suggestions on how to implement the three components of the study. A folder with all background information and curriculum schedules for each of the eight mandatory classes and optional classes was given to the participants.

Parents were informed about the study and their involvement in two ways: 1) at meetings for parents at each school at the beginning of the school year, 2) as written information, a letter and a pamphlet, which the students brought home.

At school, students were informed about the study by the school coordinator and they received written information designed specifically for them.

### Effect evaluation

#### Instruments

Measures used in the student questionnaires were mainly selected from existing smoking surveys: “Control of Adolescent Smoking” (CAS) [26,31,32], “The European Smoking Prevention Framework” (ESFA) [33,34], “Be smokeFree” (FRI) [25], “Adolescents’ lifestyle and daily living” (MULD) [2], European School Survey Project on Alcohol, and Other Drugs (ESPAD http://www.espad.org/), as well as the HBSC study [35]. For lack of suitable measures, new measures were developed for the X:IT study, specifically. Identical questionnaires for baseline and first (1FU), second (2FU), and third (3FU) follow-up surveys were used, although relevant evaluation questions were added to the follow-up versions.

#### Primary outcomes

Frequency of student smoking was measured by the self-reported question: “How often do you smoke at present?” (every day; not every day, but every week; not every week, but every month; more seldom than every month; never)(ESFA) [36]. Furthermore, questions of intentions to smoke (CAS), having smoked ever (HBSC), number of cigarettes (MULD), and age at smoking initiation (HBSC) were included in the student questionnaires.

#### Distal attitudinal influences

Ethnicity was measured as student’s and parents’ country of origin and language spoken at home (HBSC) [37]. Smoking rules at home were measured by permission to smoke at home or not (X:IT).

#### Intermediate attitudinal influences

Knowledge about smoking was measured by a selection of yes/no questions about topics mentioned in the educational material (X:IT). Evaluations of consequences were measured by motives to stay smoke-free (FRI) and perceived consequences of smoking: risk, less nervousness or loneliness, ability to stay slim (ESFA). Attitudes were measured as attitudes towards rules against smoking at school and at home (CAS), and attitudes towards peers who smoke (X:IT). Parental perception of the values of the X:IT study was measured (X:IT). Parental attitudes were measured as parents’ active engagement in their child’s smoking behaviour (X:IT).

#### Distal social influences

Family structure was measured by asking whom the students live with (Pro Children). Family socioeconomic position (SEP) was measured by parental occupation (HBSC). Availability/accessibility to cigarettes from parents and peers was measured as whether cigarettes were available in the home. Also, whether the student had ever had or bought cigarettes from their parents, peers, or others (ESPAD/X:IT). Exposure to parents’ and peers’ smoking was measured by frequency measures and by asking where parents and peers smoke (HBSC).

#### Intermediate social influences

Social relations to parents and peers were measured as frequency of physical and electronic contacts as well as number and quality of confident relations (HBSC). Social norms of parents and peers were measured, as well as the student’s motivation to comply with parents’ and peers’ norms (CAS).

#### Distal intrapersonal influences

Personal factors included gender and age.

#### Intermediate intrapersonal influences

*S*elf-efficacy for smoking was measured by questions about ability to resist smoking if friends smoke, ability to explain to others if not wanting to smoke, and ability to reject smoking if offered a cigarette [38].

#### Distal environmental influences

School level SEP was measured using a question to the school coordinator regarding the wealth of the school district (HBSC). Smoking policy in the municipality and at school was measured by questions for municipal and school coordinators. Enforcement of rules at school was measured by questions for students and school coordinators (CAS).

#### Intermediate environmental influences

Availability/accessibility of tobacco at school was measured by the possibility of buying cigarettes close to the school (HBSC). Exposure at school was measured as frequency of observed smoking among teachers and other students (CAS). Both students and school coordinators answered these questions.

#### Secondary outcomes

Additional substance use, drinking alcohol, and smoking marijuana, were measured as ever use, frequency, and initiation by student self-reports (HBSC). Life satisfaction was measured by the Cantrill ladder. Well-being was measured by questions about well-being at school and in the school class, and by questions on bullying, loneliness, and frequency of symptoms (HBSC).

### Process evaluation

Questions used for process evaluation were added to the student and to the teacher questionnaires. Questions were also added to the school and municipal coordinator questionnaires at 1FU, 2FU, and 3FU. A qualitative process evaluation was conducted where quantitative evaluation was considered to be insufficient: for the recruitment of municipalities and schools into the study (conducted spring 2010), for the implementation of the smoke-free curriculum (conducted spring 2012), and for the implementation of the smoke-free dialogue (conducted autumn 2012).

The process evaluation of the X:IT study was structured according to Steckler & Linnans’ model [39]. For the overall study and for each of the three intervention components measures of ‘context,’ ‘reach,’ ‘dose delivered,’ ‘dose received,’ ‘fidelity,’ ‘implementation,’ and ‘recruitment’ were considered, developed, and measured if considered relevant. Process data were obtained from students, teachers, and coordinators at schools and in municipalities.

#### Smoke free school premises

Context was measured by questions on smoking policy at the schools and answered by students and school coordinators. The intervention was school-based and therefore, the reach included all students attending year 7 to 9. The dose received was measured by the degree to which the schools responded to the rules. These questions were answered by both students and school coordinators.

#### Smoke free contracts

Context was measured by questions regarding parental smoking behavior. Reach was measured by the proportion of students who signed a contract.

#### Smoke free curriculum

Context was measured by questions about alternative school based initiatives aiming at reducing tobacco use (school coordinators). Reach concerned all students attending year 7 to 9. The teachers who delivered the smoke free classes answered questions about how many and which mandatory and optional exercises the classes completed. The dose received is measured by students’ reports on number and quality of lessons received. Information on fidelity was also obtained from questions to the teachers. Their focus was whether the material was easy to work with, how the material performed, whether it was relevant for the students, how the students worked with the exercises, and whether the material suited the student’s age level and level of knowledge.

Overall measures on environmental circumstances were obtained by questions to municipal coordinators regarding smoking policy and further initiatives in the municipality. Information on the procedure of recruitment for the study was determined by interviewing representatives from schools and municipalities participating in or declining to the study.

### Health economic evaluation

School and municipal coordinators provided information about resources spent in connection with the study. This included time spent coordinating the study, preparing the X:IT lessons, as well as costs for lottery prizes, textbooks and other relevant material. Data provides the basis for health economic analysis of the X:IT intervention [40,41].

### Data collection

For the baseline study, data collection among students at intervention and control schools was conducted at the beginning of year 7. Follow-up data were collected at the end of year 7 (1FU), at the end of year 8 (2FU), and at the end of year 9 (3FU). We followed the students by using information on their name, birthday, class, and school. Students who changed school were omitted from the survey. Information from signed, smoke-free contracts was collected yearly. To gain information on the curricular activities, teachers were asked to complete questionnaires on conducted mandatory and optional classes. Also, we collected data among school and municipality coordinators at each follow-up (Table 2).

**Table 2:** Data collection of the X:IT study – timeline, instrument and source of data

	***Baseline***	***Intervention***	***Follow-up 1***	***Intervention***	***Follow-up 2***	***Intervention***	***Follow-up 3***
	**September 2010**		**June 2011**		**June 2012**		**May 2013**
*Interventions schools*	Questionnaires. Students start year 7 n = 2,380		Questionnaires. Students end year 7 n = 2,202	Observation and interviews about implementation of curriculum	Questionnaires. Students end year 8 n = 1,748		Questionnaires. Students end year 9 n = 777
*Control schools*	Questionnaires. Students start year 7 n = 1,781		Questionnaires. Students end year 7 n = 1,562		Questionnaires. Students end year 8 n = 1,521		Questionnaires. Students end year 9 n = 639
*Students and parents*		Smoke free contracts n = 3,046		Smoke free contracts n = 1,731		Smoke free contracts n = 1,089	
*Study coordinator at schools*			Questionnaires n = 92		Questionnaires n = 78		Questionnaires n = 61
*Study coordinator in municipalities*			Questionnaires n = 17				Questionnaires n = 17
*Teachers at intervention schools*			Questionnaires	Interviews about implementation of curriculum	Questionnaires		Questionnaires

Students answered internet-based questionnaires in the classroom after a standardized instruction given by their teacher. The students were informed that participation was voluntary and their answers would be treated confidentially. Absentees were asked to answer questionnaires later, either at home or at school. At baseline, 4,161 students (93.1%) were included in the final data file. At first follow-up, 3,764 students were included (84.9%), and at the second follow-up, 3,269 (79.4%) were included in the data files. Due to a conflict at the Danish labor market in Spring 2013 school teachers were locked out for a month. This resulted in a response rate among students at third follow-up at only 39.4%, n = 1,416. Students were anonymized in the data file.

### Sustainability

The X:IT study is intentionally rooted in the participating municipalities to acquire sustainability. Having a municipal study coordinator, who has been following the study for years, means that the capacity is built and maintained at the municipal level and that the study can easily be implemented at other schools.

At the end of first, second, and third year of the intervention, one-day study workshops were arranged for headteachers, municipal, and school coordinators. The workshops aimed at making participants share experiences and get inspiration for new methods of teaching as well as creating an environment for discussing or solving problems that may have occurred during the intervention the previous school year. An overview of the progress of the study was also provided.

### Statistical aspects

#### Power calculations

The two Nordic studies, which the X:IT was inspired of, showed effect sizes between 30-50%. The expected effect of the intervention is a 25% lower prevalence of ‘current smokers’ (smoke daily, weekly or more seldom) at age 15 in the intervention group compared to the control group, based on what was expected to be realistic in Denmark. Other necessary assumptions for the power calculations were that each school had on average three year 7 classes with an estimated average of 20 students per class. Within schools, students’ smoking behavior is related. To estimate the intraclass correlation (0.053 for smoking weekly or more often among year 9 students), we used data from a large, nationally representative study among 15-year olds with similar measures and design, “Health Behaviour in School-aged Children 2006” (HBSC) [42]. Using these assumptions and a power of 80%, we needed 46 intervention schools and 46 control schools. Power calculations were conducted according to Donner & Klar, [43].

#### Data process and analyses

Baseline data were imported into SAS version 9.2. Information from smoke-free contracts was merged with the baseline data. Variables on ethnic background were categorized into ethnic Danish, immigrants, and descendents of immigrants according to the definitions by Statistics Denmark. Variables on parental occupation were coded into six groups according to the Danish National Institute of Social Research: social class I (high) to V (low) and a group VI covering parents who were living on social welfare benefits. The students were categorized, according to the highest ranking parent, into three groups, family SEP I-II (high), III-IV (medium), and V-VI (low).

Future analyses of first follow-up data will be conducted by means of multilevel logistic regression models. Furthermore, available case and intention-to-treat analyses will be performed; the latter with multiple imputation of missing data. In the analysis of second follow-up, a mixed model with repeated measures will be used. The random effects will handle the covariance within schools and classes. The fixed effects will include an interaction between time and the intervention indicator.

### Ethical issues

There is no formal institution for ethical assessment and approval of questionnaire-based population studies in Denmark. When inviting the schools to participate, head teachers received written information about the study. Students and their parents were informed about the study. They were informed that participation was voluntary, that their information would be used for research purposes only and treated confidentially. Parents were informed of the possibility of having their child withdrawn from the data base. The study is registered at the Danish Data Protection Agency, ref: 2010-54-0930.

## Results

### Process evaluation of the recruitment

Semi-structured interviews with schools (n = 3) and municipalities (n = 5), who agreed to participate and schools (n = 3), and municipalities (n = 5) who declined to participate were performed to assess possible bias of participation. Generally, schools had three main reasons not to participate in the study. First, schools found that they lacked time or resources and could not engage in any more studies. Second, some schools had problems fulfilling the smoking restrictions of the study, especially for the teachers and therefore chose to decline the invitation. And third, for some schools, headteachers considered that putting smoking on the agenda seemed irrelevant because the students at the school had larger problems than those of smoking. Other schools believed they had few or no students who smoked. Smoking was, therefore, not considered a problem.

The main reason for municipalities not to participate in the study was a common worry that schools in the municipality were overloaded with work. Hence, the staff at the municipalities was reluctant to impose their schools further obligations. Some considered the study to be too large, carrying too many demands or the role as school coordinator to be overwhelming. Moreover, some argued that they could not ask their schools to be randomly selected to intervention and control schools. A number of municipalities prioritized alternative health promotion initiatives.

### Descriptive analyses of baseline data

Ninety-seven schools from 17 municipalities entered random allocation. After allocation, three schools withdrew from the study leaving 51 schools as intervention schools and 43 schools as control schools. At baseline in September 2010, 4,468 year 7 students were eligible, of which 4,167 answered the baseline questionnaire (response rate = 93.3%). We had to exclude six questionnaires due to sabotage, leaving 4,161 students in the final data file. Intervention schools and control schools did not differ by average number of year 7 students per school. Further, there were no differences in the gender composition or the composition of ethnic background, own smoking status or whether smoking control was enforced at school premises (Table 3). In the control group, more students were from high socioeconomic position compared to the intervention group, whereas, students from lower SEP families were equally distributed. More students at control schools saw teachers smoke daily and more parents in the intervention group were daily smokers. More students from intervention schools answered correctly to questions regarding knowledge of smoking and tobacco (Table 3).

**Table 3:** Description of the intervention and control group at baseline in the X:IT study

	***Intervention group***	***Control group***
*Background factors*		
Number of schools (%)	51 (54.3%)	43 (45.7%)
Number of students (%)	2380 (57.2%)	1781 (42.8%)
Average number of students pr. school	49.6	45.0
Sex (boys) (E)	50.8%	51.3%
Ethnic background (A)		
• Danes	93.0%	92.8%
• Descendants	4.1%	4.2%
• Immigrants	2.9%	3.1%
Family socioeconomic position (SEP) (C)		
• High	33.6%	38.7%
• Medium	48.4%	42.8%
• Low	18.0%	18.5%
*Intermediate factors: individual*		
At least 7 out of 13 right answers on knowledge questions (B)	60.1%	54.2%
Students should be allowed to smoke outdoors on school premises (B)	12.8%	13.8%
Difficult to stay smoke-free if friends smoke (F)	59.9%	60.2%
Willing to comply with parental nonsmoking norms (D)	90.7%	90.0%
*Intermediate factors: context*		
Allowed to smoke at home (A)	4.7%	4.1%
Parents daily smokers (C)		
• Male	31.1%	27.9%
• Female	28.7%	24.3%
Best friend smokes (C)	6.9%	8.3%
Teachers control smoking (G)		
• Daily	6.4%	6.0%
• Sometimes	27.7%	25.2%
• Don’t know	47.1%	49.3
See teachers smoke (H)		
• Daily	17.2%	20.5%
• Sometimes	43.4%	43.1%
*Outcome*		
Have ever tried smoking	17.4%	19.2%
Monthly smokers	3.0%	4.2%

The letters A-H refer to factors in the influential streams in Figure 1.

## Discussion

At the beginning of year 7, almost one fifth of the students in the X:IT study had tried to smoke, but only a small proportion of students smoked regularly. More than 60% of the students saw teachers smoke sometimes or every day during school hours. However, only one third of the students witnessed teachers checking student smoking on school premises. The prevalence of parental smokers was high (24.3% to 31.1%) but are consistent with the prevalence among the adult Danish population of the relevant age group [44]. The X:IT baseline study thereby support earlier studies [2,4] indicating a remaining need for interventions aimed at adolescent smoking in Denmark.

The X:IT intervention was inspired by the social influences approach. The intervention included three elements: 1) a strict anti-smoking school policy for students and teachers requering no visible smoking during school hours, 2) parental involvement including commitments for the student not to smoke, by signing contracts between students and a significant adult each year for three years as well as smoke-free dialogues, and 3) a ‘smoke-free’ curriculum including information about short and long-term health consequences of smoking, the benefits of staying smoke free, rates of smoking among Danish adults and adolescents, trainings on media and commercial influences, and skills training of competences to resist direct and indirect pressure to smoke.

A review study by Cuijpers [45] recommended the following ingredients to be included in order to achieve effective school-based drug prevention programs: 1) interactive delivery methods, 2) use of the social influence model, 3) focus on norms, and commitment and the intention not to use, 4) community interventions, e.g. family involvement, 5) use of peer leaders, and 6) inclusion of skills and practice in the use of refusal [45]. Apart from the use of peer leaders, the X:IT intervention included all the above mentioned ingredients.

X:IT was designed as a multi-modal program including initiatives within and beyond the school. The initiatives aim at strengthening and enforcing the rules for smoking at school premises beyond the scope of the Danish legislation. Also by including parents by way of signing smoke-free contracts [24]. In a Cochrane review of school-based programs for preventing smoking, Thomas & Perera [9] included randomized controlled trials and multi-modal programs, only. Nine out of thirteen studies drawing on social influence models found smoking prevalence to decline. The authors concluded that the evidence is still too weak to recommend which components should be included in a multimodal study for tobacco prevention [9]. Flay [46] criticized the rigid criteria used in the study for papers, in order to be included in the above mentioned Cochrane review. He claimed that many high quality studies were excluded from the review which limits the conclusions regarding effectiveness of school-based smoking prevention programs. Flay summarized results from meta-analyses, which had been more inclusive. He concluded that, school-based programs can have significant long-term effects if they: 1) use interactive social influences or social skills programs; 2) involve 15 or more lessons some of them encompassing year nine; and 3) produce substantial short-term effects [46].

Earlier antismoking studies, which focused on interventions aimed at adolescents from lower socioeconomic backgrounds, used social influences and social norms [47]. They also targeted the parents and recommended smoking prevention policies [48] as effective tools in preventing smoking among this group of adolescents. The X:IT intervention included all of these elements and consequently, created a basis for studying social inequality in smoking interventions.

Municipalities and schools participating in X:IT were evenly distributed across Denmark, which makes X:IT a nationwide study. All Danish municipalities and schools were invited to participate, but they had to register actively to join the study. This procedure may have introduced selection into the study. It may be schools with exceptional resources or specific engagement that have registered. On the other hand, smoking cluster in schools and is more prevalent among socially disadvantaged children. This could imply a stronger incentive for some schools to join the study. After registering for the study, schools were randomly allocated to either intervention or control.

Analyses did not reveal systematic differences between the intervention and control group. Randomization guarantees equal distribution of covariates across intervention and control groups. An observed imbalance can happen in any randomization, but a statistical significant distribution is unlikely to influence the effect of the intervention. Therefore, homogeneity should not be tested [49].

Power calculations took into account the multilevel structure of students nested in schools, which was the level of randomization. At baseline there was no significant attrition as more than ninety percent of the enrolled students answered the baseline questionnaires in both the intervention and the control schools.

All variables in the evaluation of the X:IT intervention were based on self-reported answers. Studies which examined the validity of adolescent self-reported smoking against biochemical measures found high sensitivity and specificity [50,51]. Even so, irregular smoking and timing of recent smoking influence the validity [52,53]. This indicates that questionnaires seem to provide reasonable estimates of the prevalence of adolescent smoking.

Measures in the X:IT study are mainly based on measures used in international validated studies, ESFA [33,34], and HBSC [37,54,55]. Measures developed for the X:IT study have been tested for face validity in the pilot tests, but are not validated any further.

In the X:IT study, we want to examine the overall effect of the intervention. Previous intervention studies either did not measure the implementation of the interventions or measured implementation by use of single items [56,57]. The evaluation of X:IT measured several characteristics of the implementation [39] for each of the three main components of the intervention. Quantitative measures of the implementation were used. This will improve the ability to account for the degree of implementation of the intervention components when interpreting the study. We therby hope to add to the literature on implementation research of multi-component smoking intervention studies.

The literature on socially differential effects of smoking interventions is sparse, especially when investigating interventions among adolescents [58,59]. We want to use the X:IT evaluation to examine the socially differential effects of our intervention: Does it have equal effects among school children from both high and low socioeconomic positions or does it widen the socioeconomic gap in smoking behavior?

Finally, if the X:IT intervention proves to be effective and reasonable easy to implement; the strategy of anchoring the study in the Danish Cancer Society and in municipalities should increase its sustainability. Municipalities are in close contact with the schools and can offer the study to all schools in their district.

## Conclusions

The X:IT intervention was a large, theory based, multi-component school-based intervention evaluated by a randomized trial. Its aim is reducing adolescent smoking by 25% during a three-year intervention period. The intervention included three main components: 1) smoke-free school premises, 2) parental involvement including smoke-free dialogues and signing of smoke-free contracts between student and parent, and 3) a curriculum addressing the issues of smoking. Meta-analyses have confirmed components to be efficient, also in a Nordic setting. The X:IT intervention filled a gap in Denmark. It was evaluated by a large, randomized trial with thorough measurements and performance of process-, effect-, and health economic evaluations of the study. We collect quantitative and qualitative data from students and study coordinators at schools and in municipalities at baseline, first, second, and third follow-up. We collect data meticulously on the implementation of the components. This enables us to add to the literature on quantitative measurements of degree of implementation. It also enables us to take into account the degree of implementation when estimating the effectiveness of the intervention. Furthermore, as the X:IT intervention was school-based, it has the potential to reach all children from both higher and lower socioeconomic backgrounds, a potential which provides the opportunity for studying social inequality in the effect of the smoking intervention.

## Competing interests

The authors declare that they have no competing interests.

## Authors’ contribution

AA is the principal investigator of the evaluation of the X:IT intervention. AA coordinated the evaluation, acquisition of data, statistical analysis, and interpretation of data and drafted the manuscript. LSB participated in collection of data, procession of SAS data files, and revised the manuscript critically. LWR and LW are principal investigators of the development and design of the X:IT intervention. LWR contributed to the design, coordinated the implementation, and revised the manuscript critically. LW participated in developing the design, implementation of the study, developed the teaching materials, and revised the manuscript critically. PDJ participated in the design and implementation of the study, and revised the manuscript critically. MS participated in collection of data, procession of SAS data files, and revised the manuscript critically. PD participated in the design, and revised the manuscript critically. PDU acquired the funding, participated in the design of the evaluation, supervised the project group, and revised the manuscript critically. All authors read and approved the final manuscript.

## Authors’ information

The X:IT study is a collaboration between Centre for Intervention Research in Health Promotion and Disease Prevention and the Danish Cancer Society. Researchers from the Danish Cancer Society developed and implemented the project and researchers from Centre for Intervention Research conducted an independent and impartial scientific evaluation of the study.

The X:IT study is registered at Current Controlled Trials ISRCTN77415416.

## Pre-publication history

The pre-publication history for this paper can be accessed here:

http://www.biomedcentral.com/1471-2458/14/518/prepub
